# Temporal changes in fecal indicator bacteria and potential waterborne pathogens at Sequiota Spring: from sewer to spring

**DOI:** 10.3389/fmicb.2025.1607684

**Published:** 2025-10-29

**Authors:** William J. Durstock, Marc R. Owen, Babur S. Mirza

**Affiliations:** ^1^Department of Biology, Missouri State University, Springfield, MO, United States; ^2^Ozarks Environmental and Water Resources Institute, Missouri State University, Springfield, MO, United States

**Keywords:** non-point source pollution, microbial source tracking, waterborne pathogens, bacterially impaired freshwater spring, sewer infrastructure repair

## Abstract

Waterborne pathogens from human fecal material pose significant health risks in karst environments, where water can easily infiltrate springs, rivers, and streams via old, leaky septic tanks and damaged sewer lines. We collected 24 samples over three summers and one winter to monitor fecal indicator bacteria (FIB) and potential waterborne pathogens in Sequiota Spring using Microbial Source Tracking (MST) and Illumina paired-end sequencing of bacterial 16S rRNA gene amplicons. MST indicated a significant reduction (46 times) in human fecal indicator bacteria (HFIB), particularly *Bacteroides dorei*, from 2020 to 2022. Similarly, 16S rRNA gene sequencing showed a decline in *B. dorei* sequences, from 56% of all *Bacteroidetes* sequences in 2020 to just 4% of all retrieved *Bacteroidetes* sequences in 2022. Furthermore, 16S rRNA gene sequences within the *Enterobacteriaceae* and *Arcobacteraceae* families, related to the genera *E. kobei* and *A. cryaerophilus* also showed a decline after repair work. In contrast, sequences related to *Legionella*, remained consistent throughout the study. Winter 2019 HFIB levels were similar to summer 2019, indicating comparable pre-repair contamination. Waterfowl-associated FIB remained low (~300 cells/L) and stable from 2019 to 2022, suggesting that sewer repairs were the primary driver of HFIB reduction. These results suggests that repairing old sewer infrastructure substantially reduced human fecal contamination and decreased the presence of potential waterborne pathogens, improving water quality. This study highlights the effective application of molecular techniques under field conditions in identifying and addressing nonpoint source human fecal contamination at Sequiota Spring.

## 1 Introduction

Freshwater resources are a major source of public drinking water supplies and provide vital ecological services (Jackson et al., 2001; Baron et al., 2002; Environmental Protection Agency, 2024a). Bacterial contamination of freshwater resources is a significant concern to public health. According to the US Environmental Protection Agency (Environmental Protection Agency, 2004), up to 23% of freshwater rivers and streams in the USA have been classified as bacterially impaired due to the high abundance of *Enterococci* and fecal coliforms originating from point and non-point sources of pollution (Environmental Protection Agency, 2017). Springs, which are a part of freshwater resources and support diverse aquatic ecosystems, are also facing contamination threats from point and non-point sources of fecal contamination (An and Breidenbach, 2005; Pronk et al., 2007).

Freshwater springs in the Ozarks Mountains deliver millions of gallons of water to various rivers in Missouri, contributing significantly to the region’s hydrology (Vineyard and Feder, 1982; Wright Water Engineers, 2001). Every year, thousands of people visit these springs for recreational purposes, drawn by their natural beauty and potential for outdoor activities (Crisler and Hunt, 1952; Vineyard and Feder, 1982). In addition to serving as recreational hubs, these springs and underground water caves host a diverse array of aquatic species, including numerous species of fish and aquatic invertebrates (Berner, 1951). Several of these species, such as the Ozark Hellbender (*Cryptobranchus alleganiensis*) or the Grotto Sculpin (*Cottus specus*), are endangered and endemic to Missouri. Clean water is crucial for the survival of these species and to aquatic invertebrates, which are vulnerable to changes in water quality (Burgmeier et al., 2011; Diaz et al., 2020).

Freshwater springs within the karst environment have a history of being contaminated with domestic waste due to the highly permeable nature of karst environments (Kačaroğlu, 1999; Pronk et al., 2007; Zhang et al., 2014). Contaminants from the surface can easily penetrate into the underground water, causing potential health risks associated with tourism and posing a direct threat to endangered aquatic species (Zhang et al., 2014; Environmental Protection Agency, 2024c). Pollution entering underground water systems can originate from both point and non-point sources, such as wastewater treatment facilities (Gücker et al., 2006; McCance et al., 2020), broken sewer lines (Bishop et al., 1998; Reynolds and Barrett, 2003), leaking septic tanks (Katz et al., 2011; Withers et al., 2014), runoff from agricultural farms, manufacturing waste, and various other industrial sources (Kačaroğlu, 1999; Boyer and Pasquarell, 1999; Buckerfield et al., 2020; Lee S. et al., 2020). As, human fecal contamination is a primary health concern because fecal material of infected individuals carries high loads of potential waterborne bacterial pathogens. Levels can be up to 10^10^ *Salmonella* cells and 10^9^ *Shigella* cells per gram fecal material of an infected individual (Gerba, 2015; García-Aljaro et al., 2018). Hence, one of the main focuses of the current study was to identify and remediate the sources of fecal contamination in Sequiota Spring. Sequiota Spring is associated with Sequiota cave which serves as a recreational park and a habitat for many aquatic birds.

This study was conducted in collaboration with the Environmental Services Department (ESD) of the City of Springfield to monitor the extent of human fecal contamination in Sequiota Spring. The focus was on assessing contamination levels in relation to the repair of sewer lines upstream in the urbanized portion of the recharge area for Sequiota Spring. Previous studies (Elmund et al., 1999; Kinzelman et al., 2005; Buckalew et al., 2006; Lee et al., 2014) consistently detected the presence of *E. coli* and fecal coliforms using the IDEXX Colilert testing method. The current study monitored fecal contamination from human and waterfowl sources over 3 years, before and after the ESD’s remediation efforts to reduce non-point source pollution. We also evaluated changes in potential waterborne pathogens using high-throughput sequencing of 16S rRNA gene amplicons during the same period.

The primary objectives of the study were: (i) to monitor human fecal contamination levels over time, before and after replacing or lining of old vitrified clay sewer pipes, and (ii) to assess changes in the distribution of potential waterborne pathogens in Sequiota Spring associated with the repair of leaky sewer lines.

## 2 Materials and methods

### 2.1 Site description

**Figure 1:**
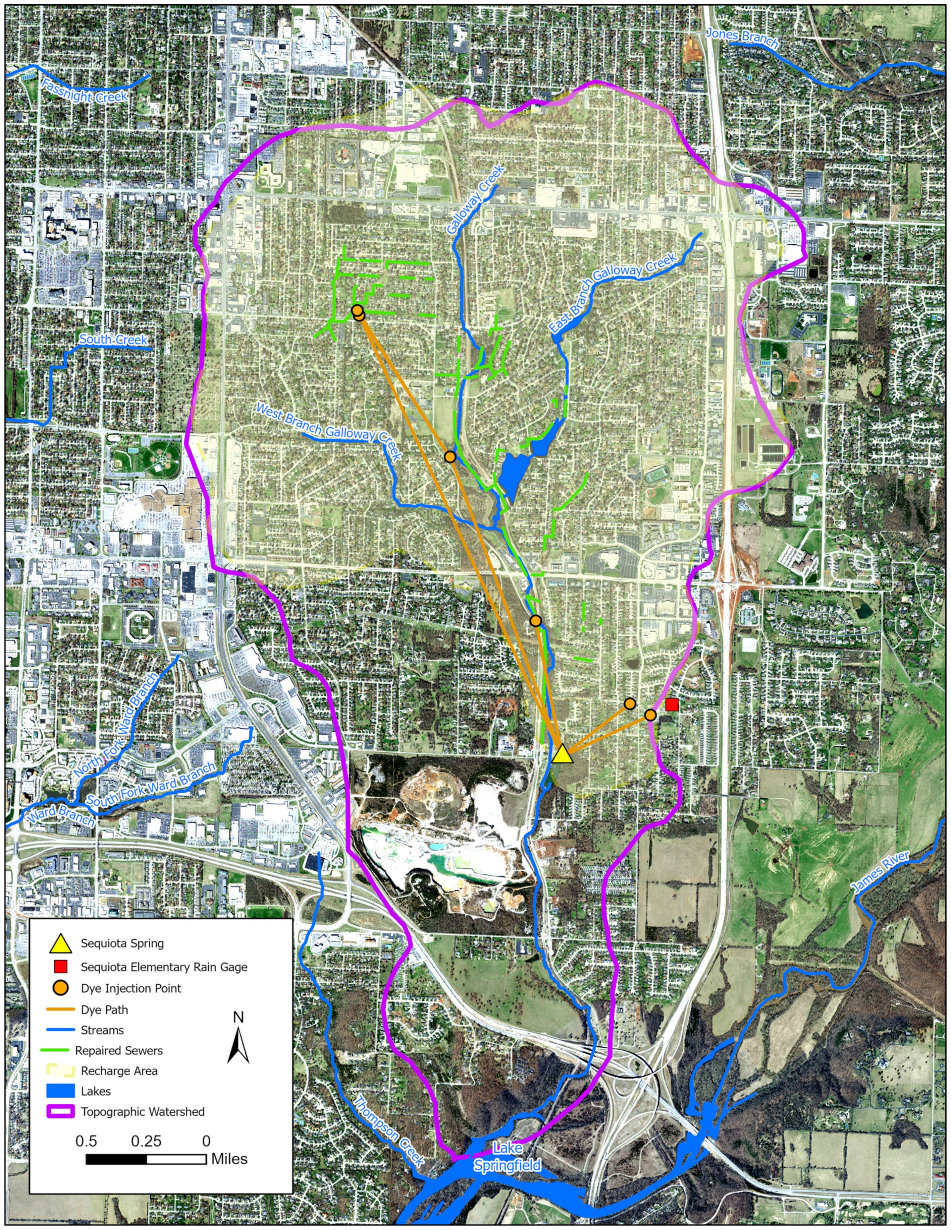
Map of the Sequiota Spring sampling site and its recharge area. Green lines indicate sewer lines that were replaced or repaired. A total of 24 water samples were collected from the spring: 18 samples collected before or during the repair work, and six samples after the repair. Orange lines represent flow paths identified in previous dye-tracing studies.

Sequiota Spring is located in southeast Springfield Missouri. There is a cave associated with Sequiota Spring Park. The cave is mostly limestone and approximately 1,000 ft. deep (Vineyard and Feder, 1982). The Sequiota Spring acts as an outlet for the Galloway Watershed (Figure 1) with an area of approximately 12.5 km^2^, and the pond is a summertime habitat for waterfowl. Previously (1986–2021), Sequiota Spring showed presence of *E. coli* and fecal coliform (Vineyard and Feder, 1982; Owen et al., 2021; Kincaid et al., 2022). We temporally monitored water samples from the Sequiota Spring for human and waterfowl associated fecal bacteria and distribution of potential waterborne pathogens before and after the remediation work. Waterfowl fecal indicator bacteria were monitored as a control because if human fecal bacteria are linked to leaky sewer lines, then following repair work, we would expect a reduction in human fecal bacteria. However, waterfowl fecal indicator bacteria should remain persistent over time, as they are consistently present during the summer.

### 2.2 Water collection and processing

Overall, a total of 24 water samples were collected during summers (2019, 2020, and 2022) and winter (2019) using sterile 5-gallon polypropylene carboys and transported back to the laboratory on ice. At each sampling time, six replicate water samples were collected across two events, with three of the replicates taken 2 weeks apart (Table 1). This design provided both replication within each year and temporal resolution across sampling events. In total, 18 samples were from summer collections (2019, 2020, and 2022), and 6 samples were from winter 2019. The water sample collection and processing details were the same as described previously (Kincaid et al., 2022). A detailed description of sampling dates, time, number of replicates, volume of water filtered, *E. coli* concentrations measured by IDEXX, sequencing depth, and rainfall data are reported in Table 1.

**Table 1:** Sampling details for Sequiota Spring across 2019–2022, including date, time, number of replicates (*n*), volume of water filtered, *E. coli* concentrations measured by the IDEXX method (MPN/100 mL), number of 16S rRNA gene sequences retrieved, and cumulative rainfall over the 10 days preceding sampling.

Date	Time	*n*	Water volume filtered (L)	IDEXX (MPN/100 mL)	Number of sequences	10-day rain (in)
June 13, 2019	14:15	3	1.75	921	63,484	1.1
June 28, 2019	14:00	3	2.0	488	73,896	3.5
Nov 18,2019	14:00	3	1.5	1,203	81,169	0.1
Dec 5, 2019	14:00	3	1.5	548	125,627	0.9
July 7, 2020	12:30	3	1.25	1,120	88,408	0.4
July 16, 2020	11:30	3	1.5	2,827	56,670	0.0
June 21, 2022	13:15	2	1.5	67.2	102,774	0.0
June 28, 2022	13:35	4	1.5	46.2	329,285	0.0

### 2.3 DNA extraction and sequencing

Water samples were filtered through 0.22 μm Sterivex filters (Millipore Corporation, Burlington, MA, USA) using a peristaltic pump (Masterflex, Cole–Pamer Co, Vernon Hills, IL, USA). The water filters were stored at −20 °C until further processing. Filters were then cut into small fragments using sterile scissors and placed into 50 mL tubes. Sterile water (25 mL) was added to the tubes containing fragments of filter and vortexed for 5 min to detach the bacterial cells from the filter. Suspended cells in water were harvested by centrifugation at 10,000 rpm for 5 min, and DNA was extracted using Qiagen’s DNeasy PowerLyzer PowerSoil kits (Mo Bio, Carlsbad, CA, USA). DNA was eluted with 25 μL sterile water and stored at −20 °C until further processing.

Bacterial communities from each water sample were assessed using Illumina MiSeq paired-end DNA sequencing. A two-step PCR approach was used as described in detail previously (Mayhood and Mirza, 2021). Briefly, in the first PCR, the V3–V5 region of bacterial 16S rRNA gene was amplified using primers 515F (5′-GTGCCAGCMGCCGCGG-3′) and 907R (5′-CCGTCAATTCMTTTRAGTTT-3′). These universal bacterial primers were also attached with the Illumina sequencing primers that were targeted in the second PCR amplification. Each 25 μL PCR reaction contained 1× buffer, 0.2 μM of each primer, 2.0 mM MgSO_4_, 0.2 μM of each deoxynucleoside triphosphates (dNTPs), 1.0 μL of template DNA and five units of High-Fidelity Platinum *Taq* polymerase (Thermo Fisher Scientific, Waltham, MA, USA). The conditions for the first PCR were an initial 5 min denaturation at 95 °C, followed by 30 cycles of 95 °C for 45 s, an annealing step at 56 °C for 45 s, an extension at 72 °C for 45 s and a final extension at 72 °C for 7 min. As a standard PCR procedure, we ran positive control (*E. coli* DNA) and negative control (PCR grade sterilized water) along with each set of PCR reactions. The successful amplification of PCR samples along both controls were evaluated by gel electrophoresis and staining with ethidium bromide. Amplified PCR products were cleaned using ExoSap-IT PCR Cleanup System (ThermoFisher Scientific, Waltham, MA, USA) as per the manufacture’s protocol. Cleaned PCR products of the first PCR reaction were used as the templates for the second PCR. In the second PCR, all reagents and their concentrations were the same as described above, except for the PCR primers. The primers used in the second PCR contained the Illumina sequencing adapters A and B along with the standard unique multiplex identifier sequences. The PCR conditions for the second PCR were: initial denaturation for 3 min at 95 °C, followed by 15 cycles of denaturation at 94 °C for 30 s, annealing at 60 °C for 30 s and extension at 72 °C for 30 s, with a final extension at 72 °C for 7 min. PCR products were quantified using a Nanodrop 2000 spectrophotometer (ThermoFisher Scientific, Waltham, MA, USA) and all samples were pooled together in equimolar concentrations. Pooled PCR amplicons were purified using the Agencourt AMPure beads (Beckman Coulter, Brea, CA, USA). The purified PCR products were sequenced using Illumina MiSeq paired-end DNA sequencing.

### 2.4 Sequence processing and phylogenetic analysis

The 16S rRNA gene sequences were analyzed using QIIME 2 (version 2025.7; Caporaso et al., 2010). Raw FASTQ files were imported into QIIME for quality filtering and demultiplexing. Low-quality reads were removed, and sequences were assigned to their respective samples based on unique indices. Dereplication was performed, and amplicon sequence variants (ASVs) were generated using DADA2. Taxonomic classification was conducted by comparing the sequences to the SILVA 138.1 reference database.

Representative sequences for each ASV belonging to families containing potential waterborne pathogens were further analyzed phylogenetically. These representative sequences, along with reference sequences from the respective families in GenBank, were aligned and analyzed using MEGA (version 10.1.8; Newman et al., 2016). Maximum likelihood phylogenetic trees were constructed for each family to evaluate the relatedness of the representative sequences to those in GenBank.

### 2.5 Quantitative PCR for microbial source tracking

*Bacteroidetes* specific to human and waterfowl fecal bacteria were determined using host-specific primers (Table 2). These assays were performed using the same master mix concentrations as described previously (Owen et al., 2019). Briefly, the TaqMan Universal PCR Master Mix from Thermo Fisher Scientific was used for the TaqMan assay (human marker). Each 20 μL PCR reaction contained 1× PCR master mix, 100 nM of each primer and probe, and 1 μL of template DNA. The DNA probes were modified with 6-carboxyfluorescein (FAM) as the reporter fluorophore on the 5′ end and N, N, N, N-tetramethyl-6-carboxyrhodamine (TAMRA) as the quencher on the 3′ end (Table 2). For waterfowl fecal bacteria testing, iTaq Fast SYBR Green Supermix with ROX (Bio-Rad, Inc., Hercules, CA), 100 nM of each primers, and 2 μL of template DNA were used. PCR conditions were as follows: 94 °C for 2 min, followed by 40 cycles of denaturation at 94 °C for 30 s, annealing at 58–60 °C (depending on marker gene) for 1 min, and extension at 72 °C for 30 s. PCR-grade water was used as a negative control. The specificity of the qPCR products for SYBR Green Supermix was confirmed by melting curve analysis.

**Table 2:** Primers and probe for qPCR.

Primer	Sequences	Marker	Reference
F-HF183	F 5′-ATC ATG AGT TCA CAT GTC CG-3′	Human	Layton et al. (2006)
R-SSHBacR	R 5′-TAC CCC GCC TAC TAT CTA ATG-3′	*Bacteroides dorei*	
SSHBAC-PRB	(FAM)-TTAAAGGTATTTTCCGGTAGACGATGG-(TAMRA)		
CGPrevf5-F	F 5′-CCC ACC AAG CCG TCG AT	Canadian Goose	Lu et al. (2009)
CGPrevf5-R	R 5′-GCT TAA CCT GCG GCC TG	*Prevotella* spp.	
GFD-F	F 5′-TCG GCT GAG CAC TCT AGG G	General waterfowl	Green et al. (2011)
GFD-R	R 5′-GCG TCT CTT TGT ACA TCC CA	*Helicobacter* spp.	

For both TaqMan and SYBR Green qPCR, a standard curve was generated from serial dilutions (10^1 to 10^9) of plasmid DNA containing the specific marker gene. qPCR efficiency (E) was calculated using the equation: *E* = 10[−1/slope]. The absolute quantification of the targeted gene was performed by interpolating Ct values (cycle threshold value) of unknown samples from the standard curve, which was prepared with serial dilutions of known quantities of plasmid DNA inserted with the targeted gene (human and waterfowl-specific bacterial markers). Details on the standards preparation procedure and quantification have been previously reported (Mirza et al., 2017).

The qPCR primer and probe combinations used in this study have been well-tested and optimized for the specific amplification of bacterial marker genes from human (Seurinck et al., 2005; Harwood and Stoeckel, 2011) and waterfowl (Green et al., 2011) fecal materials. The positive standard DNA material (plasmid with inserts of specific marker genes) was used as reference material for our unknown water samples. Negative samples (sterile water) showed no amplification. The regression line of the standard curve were generated through serial dilutions of specific marker genes. It is important to note that during the summer and winter of 2019 and the first sampling of 2020, we used PCR primers specifically designed to identify goose fecal contamination. However, for the second sampling of 2020 and both sampling events in 2022, we used broader waterfowl-specific PCR primers capable of detecting fecal contamination from various bird species, including ducks, swans, and geese. This change was made after observing the presence of swans during the summer of 2020.

### 2.6 Statistical analysis

We analyzed the overall bacterial community using the Bray–Curtis dissimilarity matrix. For NMDS analysis (Non-metric Multidimensional Scaling), the data were log-transformed. The significance of diversity among bacterial communities at different time points were assessed using Analysis of similarity (ANOSIM). NMDS analysis was conducted in RStudio software 4.4.1 (R Development Core Team, 2024; Rstudio Team, 2024) using the vegan, readxl, and ggplot2 (data visualization) packages. A Bray–Curtis dissimilarity matrix was created using the vegdist() function, and NMDS was performed with metaMDS() from the vegan package.

## 3 Results and discussion

We quantified the abundance of human- and waterfowl-specific fecal marker bacteria using qPCR, and distribution of potential waterborne pathogen*s*, including members of the *Bacteroidaceae*, *Enterobacteriaceae*, *Arcobacteraceae*, and *Legionellaceae* families, using high-throughput DNA sequencing.

### 3.1 Microbial source tracking and *E. coli* quantification

**Figure 2:**
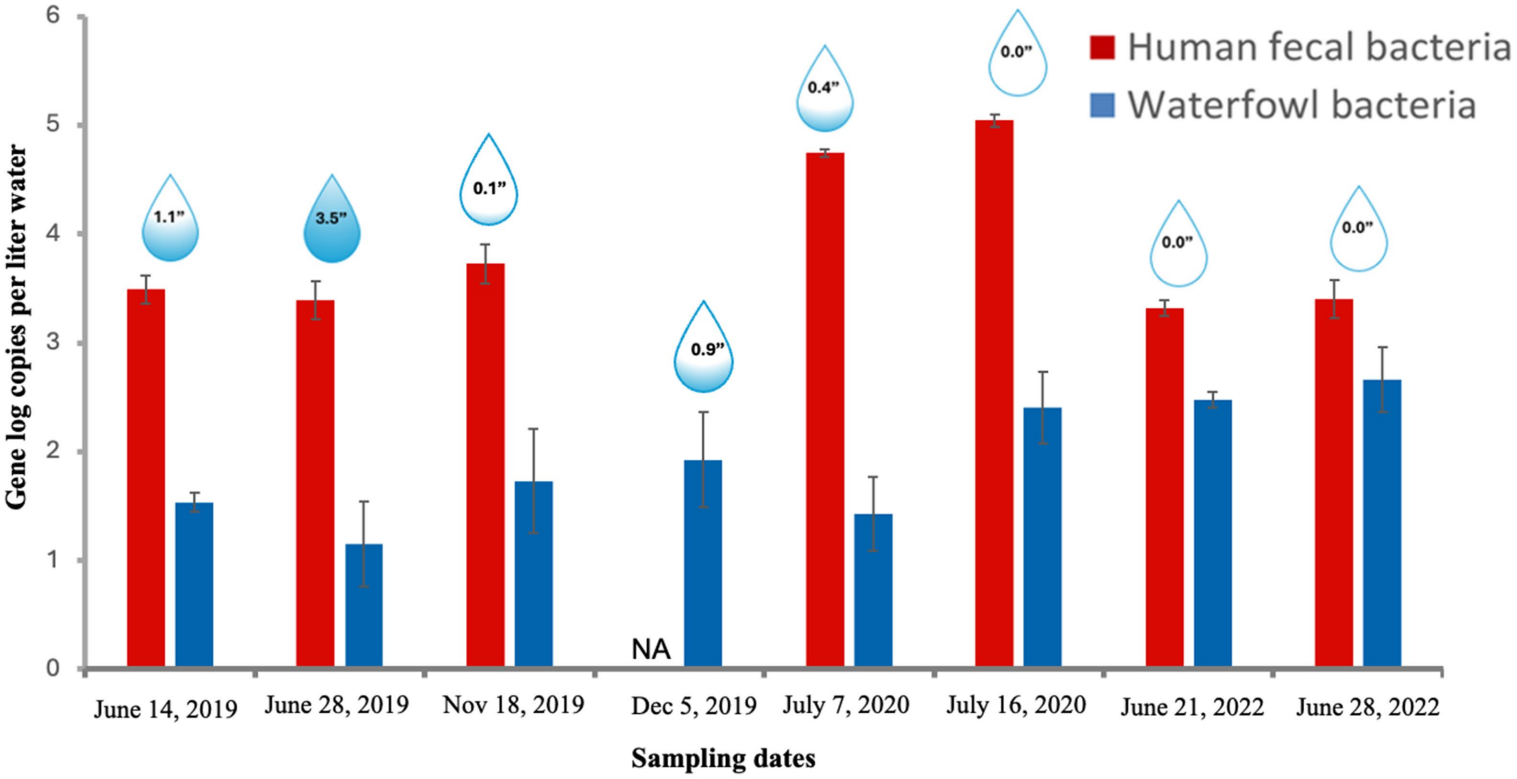
Abundance of human and waterfowl fecal indicator bacteria (log copies/L) quantified using qPCR. Raindrop icons indicate the inches of rainfall within 10 days prior to sampling. Each bar represents the mean of three replicates, with error bars indicating the standard error. NA, not available.

The qPCR analysis indicated the presence of both human and waterfowl fecal bacteria in Sequiota Spring water samples collected over three summers and one winter (Figure 2). The regression line for the standard curve, generated through serial dilutions of plasmids containing the host-specific markers, showed coefficients of determination of 0.995 and 0.997 for the human and waterfowl markers, respectively. The PCR amplification efficiencies ranged from 91% to 96%. Since SYBR Green dye was used for the waterfowl marker, we confirmed amplicon specificity through melting curve analysis, which indicated a single peak.

We observed a significant variation in human fecal indicator bacterial (HFIB) copy numbers across different sampling times (Figure 2; *p* < 0.05). HFIB levels were significantly higher in 2020 (83,157 ± 3,719 gene copies/L; mean ± SE) compared to 2019 summer (2,886 ± 465 gene copies/L) and winter of 2019 (5,622 ± 1,196 gene copies/L). After 2020, HFIB levels declined, returning to pre-2020 levels (2,387 ± 324 gene copies/L in 2022). HFIB numbers in 2019 (2,886) and 2022 (2,387) were not significantly different (*p* > 0.5). The observed decline in HFIB levels after 2020 could be due to the replacement or lining of 5.3 miles of old vitrified clay sewer pipes upstream of Sequiota Spring (Figure 1) by the City of Springfield. However, while the timing of HFIB declines coincides with sewer repair activities, other factors such as seasonal changes, rainfall patterns, or land-use variability could also have influenced microbial concentrations.

In contrast to high HFIB (110,685 copies/L), waterfowl fecal bacteria levels were consistently low (<528 gene copies/L) across all sampling periods (Figure 2). Despite the switch from narrow geese-specific qPCR primers used in 2019 and July 7th 2020 sampling of 2020 to more general waterfowl-specific primers in later samplings, waterfowl numbers remained low across all seasons. In the 2019 and July 7, 2020, sampling summer and winter samples using geese-specific primers ranged from 19 to 160 copies/L, while later samples from 2020 and 2022 using waterfowl-specific primers showed counts ranging from 33 to 528 copies/L. The lack of any substantial changes in waterfowl fecal bacteria levels suggests that the reduction in HFIB primarily reflects decreased human contamination (Figure 2).

In addition to host-specific qPCR markers, we quantified *E. coli* levels across sampling periods using the IDEXX method (Table 1). *Escherichia coli* abundances ranged from 488 to 1,203 MPN/100 mL in 2019, increased to 1,120 and 2,827 MPN/100 mL in the two summer 2020 samples, and declined markedly to 67 and 46 MPN/100 mL in 2022. According to U.S. EPA recreational water quality criteria for primary contact recreation, the geometric mean of *E. coli* should not exceed 126 MPN/100 mL and a statistical threshold value of 410 cfu/100 mL. By this standard, *E. coli* levels in 2019 and 2020 consistently exceeded EPA thresholds, indicating elevated health risks, while the 2022 samples fell well below the recommended limits, reflecting a substantial improvement in water quality following sewer repairs. It is also important to note that while *E. coli* serves as the regulatory fecal indicator in freshwater systems, EPA does not set numeric limits or require routine monitoring for other waterborne pathogens, such as *Legionella*, *Salmonella*, *Shigella*, *Yersinia*, etc., which were also evaluated through Illumina sequencing.

Lastly, we assessed rainfall data within the recharge area to evaluate the potential role of hydrological conditions, which are critical for understanding water quality in karst systems. In the summer 2019 samples, higher rainfall (1.1–3.5 inches/10 days) may have contributed to dilution of HFIB. In contrast, the summer 2020 samples (0.4 inches on July 7 and 0.0 inches on July 16) and both summer 2022 sampling events (0.0 inches) occurred under baseflow conditions. These patterns suggest that the observed changes in bacterial communities were most likely linked to sewer repairs rather than hydrological variability, resulting in reduced HFIB.

### 3.2 Overall bacterial community structure

**Figure 3:**
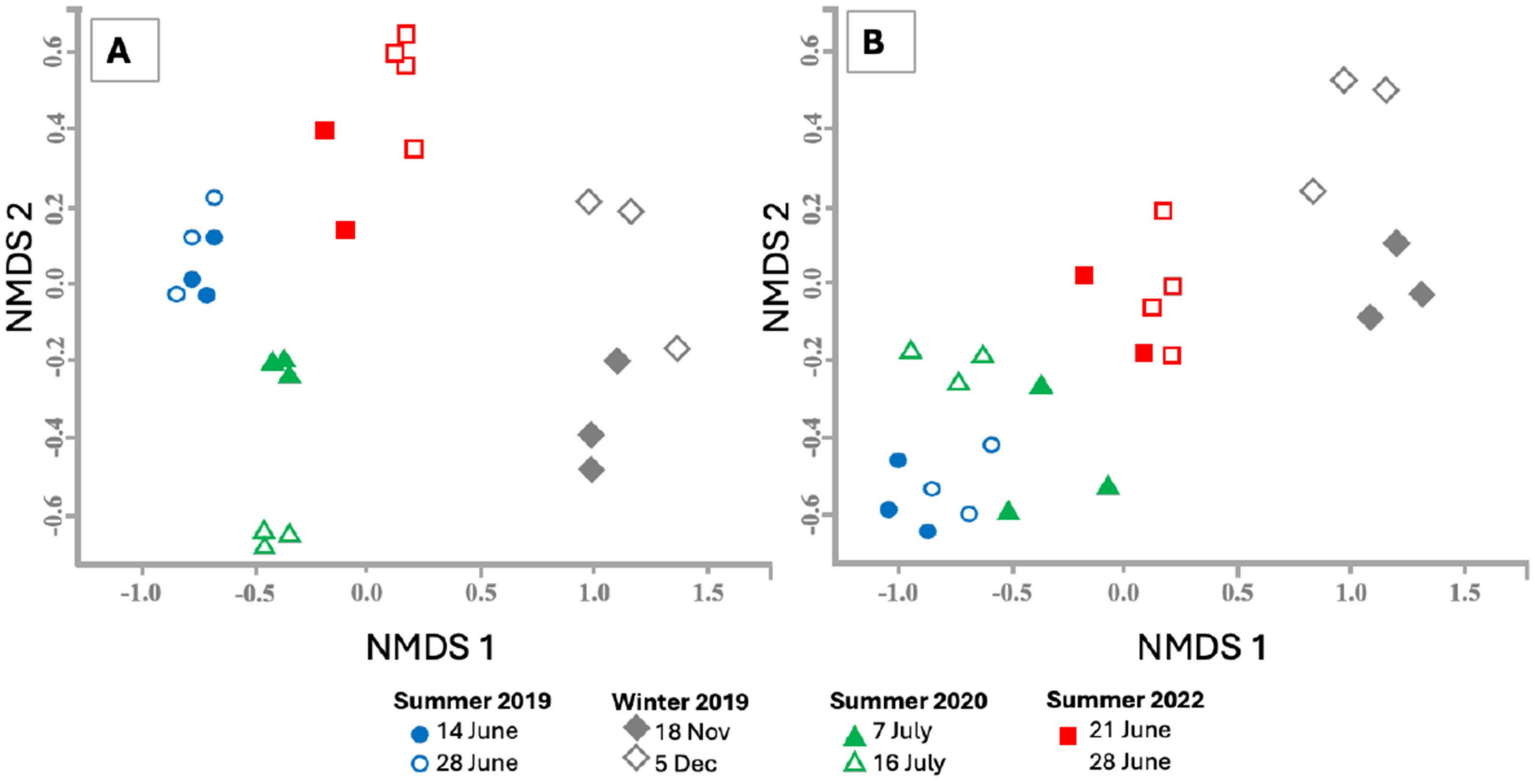
Non-parametric multidimensional scaling (NMDS) plot based on the Bray–Curtis similarity index (97% similarity) of 16S rRNA gene sequences from 24 water samples collected from Sequiota Spring. **(A)** NMDS analysis includes all 16S rRNA gene sequences, while **(B)** focuses on DNA sequences from four major bacterial families: *Bacteroidaceae*, *Enterobacteriaceae*, *Arcobacteraceae*, and *Legionellaceae*.

Overall, we retrieved 921,313 high-quality 16S rRNA sequences from 24 water samples and assessed bacterial community structure using the Bray–Curtis similarity index (97% similarity) of 16S rRNA gene sequences. Multivariate analysis (Figure 3A) revealed significant temporal variation in the distribution of bacterial communities at 97% DNA similarity across water samples. The winter 2019 samples were significantly different from all summer samples and showed greater variation within replicates than the summer samples. These differences in bacterial community structure across different years could be due to variations in water quality parameters and/or other external and internal factors. In general, microbial communities in water samples collected approximately 2 weeks apart at each location were similar, with the exception of 2020, which showed variation between samples collected on July 7 and July 14 (Figure 3A). This variation may be attributed to a rain event that occurred a few days before the July 7 sampling. We also assessed bacterial community structure based on 16S rRNA gene sequences related to four families: *Enterobacteriaceae*, *Legionellaceae*, *Bacteroidaceae*, and *Arcobacteraceae*. Although the differences in the community structure based on the distribution of sequences from these four families were less pronounced (Figure 3B) compared to the total sequence analysis (Figure 3A), winter samples were clearly distinct from summer samples, while differences among summer samples for these families were less marked. We did not observe variation in community structure within samples collected from the same location two weeks apart based on the Bray–Curtis similarity index when considering only these four families (Figure 3B). Therefore, to simplify the information in phylogenetic analysis, we combined the number of sequences from both sampling events for each year and presented the data by year for these four families.

Overall, the temporal changes in bacterial community structure at Sequiota Spring have limited implications for broader ecological patterns within karst environments. However, the sampling design and number of samples (Environmental Protection Agency, 2024b) were sufficient to identify human fecal contamination, evaluate remediation efforts, and assess whether sewer line repairs effectively reduced contamination and potential waterborne pathogens. The primary emphasis of this study is to demonstrate how molecular tools can provide actionable information for infrastructure repair while also offering preliminary insights into the distribution of potential waterborne pathogens relative to human fecal contamination levels. The study does not directly assess the extent of human health risks associated with potential use of the site. Instead, it aims to characterize microbial community dynamics before and after repair, and to identify contamination sources in Sequiota Spring.

### 3.3 Phylum level distribution of 16S RNA gene sequences

**Figure 4:**
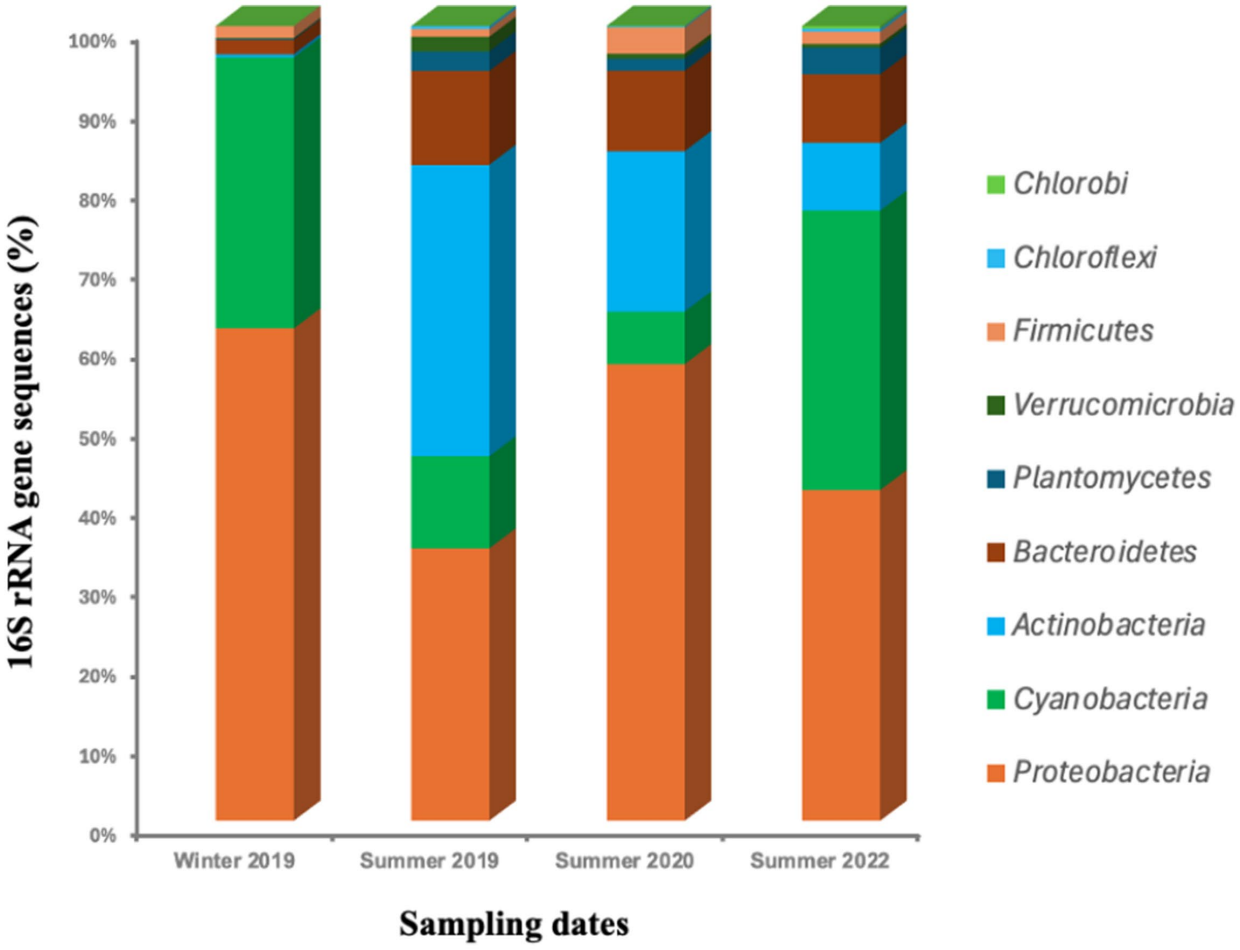
Temporal distribution of bacterial 16S rRNA gene sequences across major bacterial phyla in Sequiota Spring during the summers of 2019, 2020, and 2023 and winter of 2019. The analysis includes all 921,313 bacterial sequences obtained from 24 water samples, with each bar graph representing six replicates. Only the 10 most abundant phyla are displayed.

We classified all 16S rRNA gene sequences at the phylum level (Figure 4) and observed a variable distribution of various bacterial phyla. The most abundant were Proteobacteria (43%), Cyanobacteria (21%), Actinobacteria (16%), Bacteroidetes (8%), and Planctomyces (2%), while other bacterial phyla such as Verrucomicrobia, Firmicutes, Chloroflexi, and Chlorobi were detected at low abundance (<3%).

Proteobacteria (43%) are a diverse group of bacteria commonly found in various environments, including soil and water (Zwart et al., 2002; Kersters et al., 2006; Spain et al., 2009; Fukuyama et al., 2020). In freshwater springs, Proteobacteria can be a part of normal microflora of water or originated from outside sources such as agricultural runoff, sewage discharge, or other human activities. Proteobacteria-related sequences were more abundant in 2020 (56%) compared to their levels in 2019 (34%) and 2022 (40%). Proteobacteria-related sequences were highest in winter 2019 (61%), followed by summer 2020 (56%), summer 2022 (40%), and lowest in summer 2019 (34%).

Cyanobacteria (21%) related sequences were the second most abundant group of bacteria in water samples at Sequiota Spring (Figure 4). In contrast to Proteobacteria, Cyanobacteria-related sequences were particularly enriched in winter 2019 (34%) and summer 2022 (34%) compared to summer 2019 (11%) and summer 2020 (6%). Cyanobacteria are photosynthetic microorganisms that can thrive in freshwater environments (Whitton and Potts, 2002; Environmental Protection Agency, 2024b). The presence of cyanobacteria in Sequiota Spring was most likely linked to an upstream eutrophic pond connected via sink holes. We also observed the establishment of cyanobacterial dominated-blooms in the pond downstream of Sequiota Spring, suggesting high nutrient loading in this water source. Actinobacteria was the third most abundant bacterial phyla that was identified in the 16S rRNA gene sequences. Actinobacteria-related sequences were abundant in summer 2019 (36%) and summer 2020 (20%) but were nearly absent in winter 2019 and reduced to 8% in summer 2022. Actinobacteria are commonly found in soil and sediment environment (Waksman, 1931; Barka et al., 2015). Actinobacteria can be naturally present or introduced into water through external sources such as urban runoff, stormwater, or discharge from wastewater treatment plants, etc. (Ranjani et al., 2016; Osunmakinde et al., 2019; Lee S. et al., 2020). In addition to their role in the environment, Actinobacteria are also an important component of gut microbiome of warm-blooded animals, including humans (Barka et al., 2015).

Furthermore, we observed the presence of *Bacteroidetes*-related sequences, which are frequently detected in various environments, including soil, sediments, and the gastrointestinal tracts of humans and other animals (Thomas et al., 2011). Members of *Bacteroidetes* are commonly used as indicators of fecal contamination, highlighting potential public health risks associated with water quality (Teixeira et al., 2020). Overall, we observed that Bacteroidetes comprised 12% of sequences in summer 2019, decreased to 2% in winter 2019, and then were 10% in summer 2020 and 8% in summer 2022 (Figure 4).

### 3.4 Family level distribution of 16S RNA gene sequences

We further explored the distribution of 16S rRNA gene sequences associated with potential waterborne pathogens and fecal indicator bacteria within four families spanning three phyla: Proteobacteria (*Enterobacteriaceae* and *Legionellaceae*), Bacteroidota (*Bacteroidaceae*), and Campylobacterota (*Arcobacteraceae*) within summer samples. We did not include winter samples in the family-level comparisons, as the survival and persistence of potential waterborne pathogens in water are strongly influenced by seasonal factors such as lower winter temperatures and changes in human water use activity (Sterk et al., 2013; Levy et al., 2016). Consequently, we focused on 3 years of summer samples to provide a comparable distribution of potential waterborne pathogens before and after the repair work.

#### 3.4.1 *Bacteroidaceae*-related sequences

**Figure 5:**
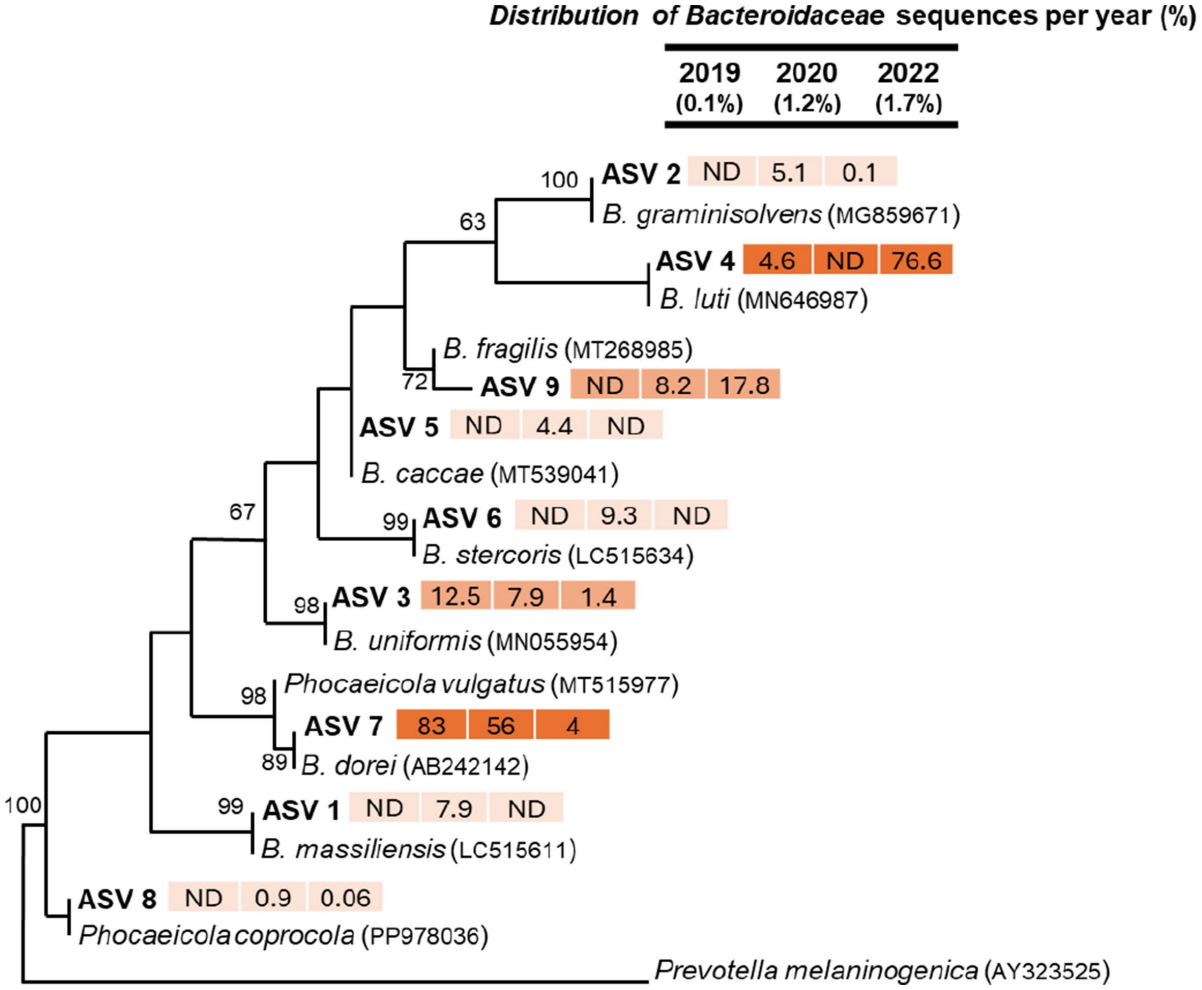
Maximum-likelihood phylogenetic tree of partial 16S rRNA gene sequences (370 bp) related to the *Bacteroidaceae* family from three sampling periods. Only ASVs with >20 sequences were included in the phylogenetic analysis. Closely related sequences from GenBank were also incorporated. Bootstrap support values above 50% are shown at the nodes. *Prevotella melaninogenica* was used as the outgroup. Numbers next to ASVs indicate the relative (%) distribution of sequences within the *Bacteroidaceae* family across different time points. The fill color of the numbers represents sequence abundance, with darker colors indicating higher sequence counts.

We identified a total of 10,109 sequences related to the family *Bacteroidaceae* across 18 summer water samples. The relative proportion of *Bacteroidaceae* sequences to total bacterial sequences increased from approximately 0.1% in 2019 to 1.7% in 2022 (Figure 5). All *Bacteroidaceae*-related sequences were grouped into 9 ASVs, and their relative distribution varied significantly across different sampling periods.

For example, 83 and 56% of the *Bacteroidaceae* sequences detected in 2019 and 2020, respectively, were clustered into ASV 7 (Figure 5). However, by 2022, only 4% of the sequences were clustered into ASV 7. The representative sequences of ASV 7 showed a close relationship to *Bacteroides dorei* sequences from GenBank which is the target of the human-specific HF183 primers, with strong bootstrap support values (Figure 5). *B. dorei* is a bacterium specific to the human gut microbiota and is frequently used as a biomarker in microbial source tracking (MST) studies to identify human fecal contamination in water resources (Bakir, 2006; Green et al., 2014; Nshimyimana et al., 2014; Ahmed et al., 2016). The observed decrease in the relative abundance of *B. dorei*-related sequences in ASV 7 from 2020 to 2022 aligns with MST results, which indicated a 14 times reduction in 16S rRNA gene sequences human fecal indicator bacteria (*B. dorei*). Apart from potential bacterial dilution due to rainfall (Figure 2), these results align with qPCR findings, which indicate a significant decrease in HFIB after repairing or replacing leaky sewer pipes. Similar to the MST results, we also detected 16S rRNA sequences identical to *B. dorei* in our amplicon sequencing data, which showed a decline in abundance in summer 2022. This provides independent validation of the MST findings.

In contrast to ASV 7, ASV 4 sequences were more abundant in summer 2022 samples than in 2019 and 2020 (Figure 5). The distribution of ASV 4 sequences accounted for 4.6% of *Bacteroidaceae*-related sequences in 2019, were undetected in 2020, and increased significantly to 76.6% in 2022. These sequences were closely related to *Bacteroides luti* from GenBank, previously isolated from anaerobic methanogenic sludge used in municipal sewage treatment (Hatamoto et al., 2014) and also detected in freshwater samples from South Korea (Lee C. et al., 2020). Considering the limited information available on *B. luti* prevalence, persistence, and ecological significance in freshwater environments, it is difficult to associate ASV 4 sequences to potential human fecal contamination.

Other ASVs were detected at lower abundances. For example, ASV 3 accounted for 12.5% of *Bacteroidaceae*-related sequences in 2019, decreasing to 7.9% in 2020 and 1.4% in 2022 (Figure 5). ASV 3 was related to *B. uniformis*, commonly found in animal feces and studied for its health relevance, particularly in rodents (Koo and Morrow, 2024; Romaní-Pérez et al., 2024). ASV 9, related to *B. fragilis*, was undetectable in summer 2019 but increased to 8.2% in 2020 and 17.8% in 2022. *B. fragilis* is a well-studied human gut bacterium associated with enterotoxigenic genes (Yu et al., 2019); however, its prevalence or potential role in freshwater systems remains unclear. Several other ASVs were mainly detected in summer 2020. ASV 1 (7.9%) was linked to *B. massiliensis*, previously isolated from a newborn’s blood (Fenner et al., 2005). ASV 2 appeared in 2020 (5%), was absent in 2019, and dropped to 0.1% in 2022. It was closely related to *B. graminisolvens*, a strict anaerobe from a methanogenic reactor treating cattle waste (Nishiyama et al., 2009), with little known about its survival in freshwater. Likewise, ASVs 5 and 6 were also exclusive to summer 2020 samples.

#### 3.4.2 Enterobacteriaceae-related sequences

**Figure 6:**
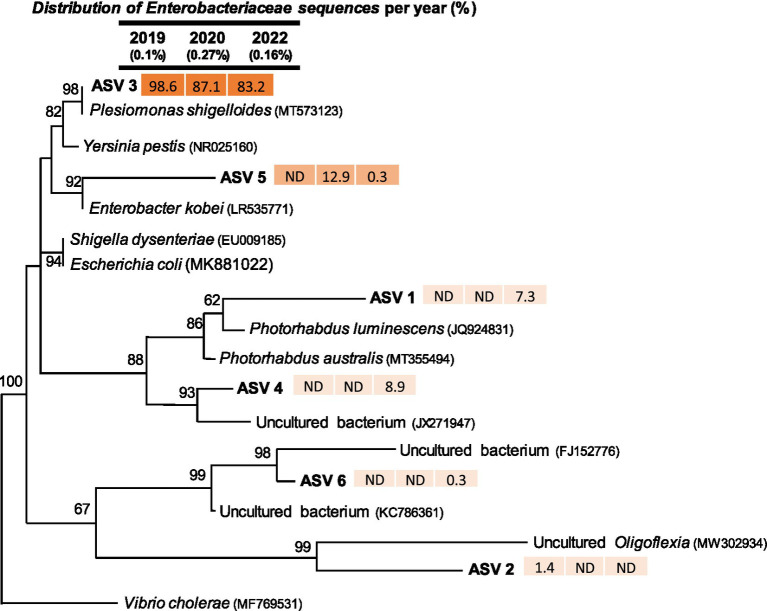
Maximum-likelihood phylogenetic tree of partial 16S rRNA gene sequences (370 bp) related to the *Enterobacteriaceae* family from three sampling periods. Only ASVs with >20 sequences were included in the phylogenetic analysis. Closely related sequences from GenBank were also incorporated. Bootstrap support values above 50% are shown at the nodes. *Vibrio cholerae* was used as the outgroup. Numbers next to ASVs indicate the relative (%) distribution of sequences within the *Enterobacteriaceae* family across different time points. The fill color of the numbers represents sequence abundance, with darker colors indicating higher sequence counts.

The *Enterobacteriaceae* family includes a diverse group of Gram-negative bacteria, with both pathogenic and non-pathogenic species. Approximately, 30% of *Enterobacteriaceae* species are considered pathogenic to humans and animals (Linton and Hinton, 1988). Overall, we retrieved 1,332 sequences related to the bacterial family *Enterobacteriaceae*. All *Enterobacteriaceae*-related sequences could be grouped into ASV1 to ASV6 (Figure 6). The relative abundance of *Enterobacteriaceae*-related sequences across different sampling times were 0.10% of all sequences in 2019, 0.27% in 2020, and 0.16% in 2022. Most of the sequences within this family were related to ASV3, which accounted for 98.6% in 2019, 87.1% in 2020, and 83.2% in 2022 of *Enterobacteriaceae*-related sequences retrieved per summer. The most closely related cultured bacterium to ASV3 was *Plesiomonas shigelloides* which is commonly found in freshwater streams, rivers, lakes, and brackish estuaries (Janda et al., 2016). A previous study Miller and Koburger (1985) also isolated *P. shigelloides* from wild-caught fish and shellfish. Generally, *P. shigelloides* is considered an opportunistic pathogen, which can cause short term gastrointestinal discomfort in humans, including stomach pain, fever, nausea, and diarrhea (Tseng et al., 2002; Janda et al., 2016). Though rare reports of disease outbreaks related to *P. shigelloides* infections have been reported, their presence in spring water suggests that further future studies should focus on its potential growth and survival of this bacteria in the environment.

The sequences in the ASV5 were closely related to *Enterobacter kobei* (Figure 6) which were non-detectable in 2019, 13% in 2020, and 0.3% in 2022. *Enterobacter kobei* belongs to a nosocomial pathogenic group, also known as ESKAPE (*Enterococcus faecium, Staphylococcus aureus, Klebsiella pneumoniae, Acinetobacter baumannii, Pseudomonas aeruginosa,* and *Escherichia coli*; Rice, 2008). Previously, *E. kobei* species have been isolated from untreated wastewater (Ludden et al., 2017) and its detection in wastewater suggests it can be present in human sewage systems. The *E. kobei* related sequences dropped from 13% in 2020 to 0.3% in 2022. *E. kobei* presence in water can be a potential concern due to their multidrug resistance (Zhou et al., 2017).

The 16S rRNA gene sequences in ASV1 and ASV4 were not detectable during the first two sampling events (2019 and 2020). However, they accounted for 7 and 9%, respectively, of the total *Enterobacteriaceae*-related sequences detected in 2022. ASV1 was closely related to *Photorhabdus luminescens*, a symbiotic bacterium associated with nematodes and known as a broad-spectrum insect pathogen (Ehlers, 2001; Duchaud et al., 2003) which can be a beneficial agent in agricultural applications (Downs et al., 2019). The sequences in ASV4 were closely related to uncultured bacterial sequences from GenBank.

#### 3.4.3 Arcobacteraceae-related sequences

**Figure 7:**
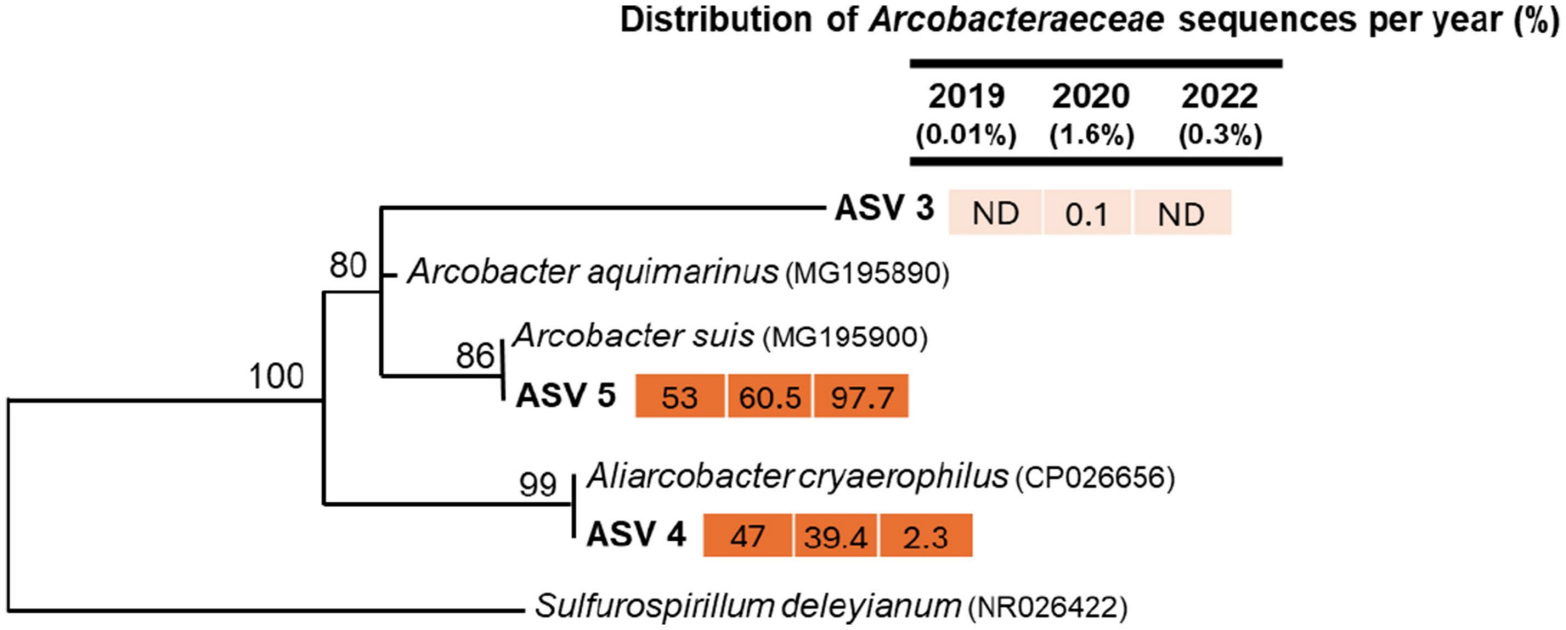
Maximum-likelihood phylogenetic tree of partial 16S rRNA gene sequences (370 bp) related to the *Arcobacteraceae* family from three sampling periods. Only ASVs with >20 sequences were included in the phylogenetic analysis. Closely related sequences from GenBank were also incorporated. Bootstrap support values above 50% are shown at the nodes. *Sulfurospirillum deleyianum* was used as the outgroup. Numbers next to ASVs indicate the relative (%) distribution of sequences within the *Arcobacteraceae* family across different time points. The fill color of the numbers represents sequence abundance, with darker colors indicating higher sequence counts.

We also assessed the relative distribution of bacterial sequences related to Arcobacteraceae (Figure 7), which can enter freshwater springs through surface runoff during rainfall, leaky sewer systems, or direct contamination from wildlife droppings (Hsu and Lee, 2015). Overall, we observed 3,761 Arcobacteraceae-related sequences, which were 0.01% in 2019, 1.6% in 2020, and 0.39% in 2022. Most of the Arcobacteraceae-related sequences were associated with ASV5, closely related to Arcobacter suis, was previously isolated from pig meat (Levican et al., 2013), buffalo’s milk (Giacometti et al., 2015), and a spinach-processing plant (Hausdorf et al., 2013). Although not much information is available on the ecology of A. suis, it has been commonly associated with food processing plants, especially porcine facilities.

The second most abundant ASV4 was most closely related to *A. cryaerophilus* and accounted for 47% of *Arcobacteraceae* related sequences in 2019 and 39% in 2020, which dropped significantly to 2.3% in 2022 (Figure 7). Typically, *A. cryaerophilus* is associated with cattle, pigs, shellfish, and poultry (Nieva-Echevarria et al., 2013; Müller et al., 2020); however, it has also been found to transmit to other animals living on farms, such as felines (Fera Figueras et al., 2009). This pathogenic species has been linked to diarrhea and bacteremia in humans (Figueras et al., 2014; Ferreira et al., 2015).

#### 3.4.4 Legionellaceae-related sequences

**Figure 8:**
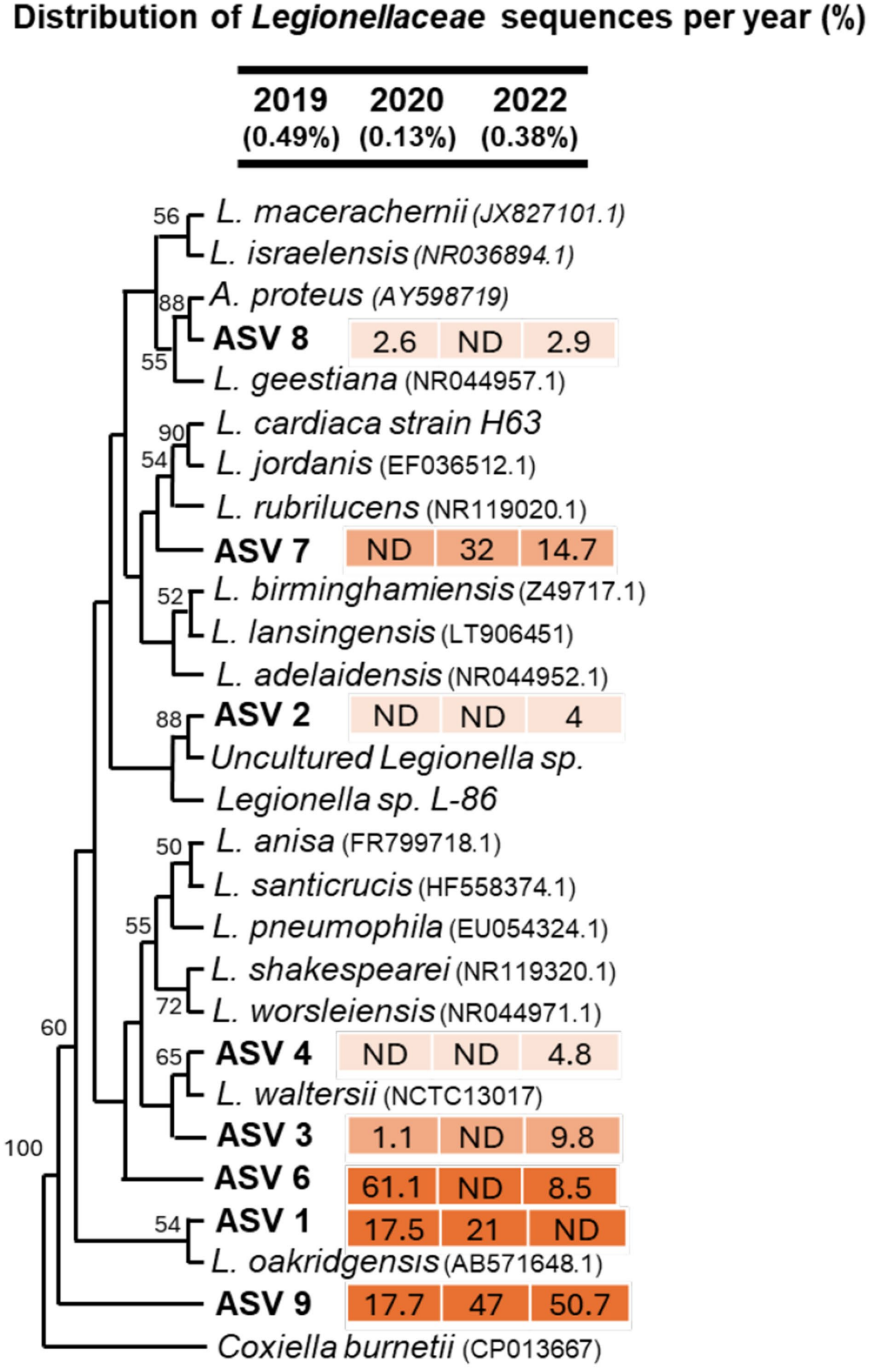
Maximum-likelihood phylogenetic tree of partial 16S rRNA gene sequences (370 bp) related to the *Legionellaceae* family from three sampling periods. Only ASVs with >20 sequences were included in the phylogenetic analysis. Closely related sequences from GenBank were also incorporated. Bootstrap support values above 50% are shown at the nodes. *Coxiella burnetii* was used as the outgroup. Numbers next to ASVs indicate the relative (%) distribution of sequences within the *Legionellaceae* family across different time points. The fill color of the numbers represents sequence abundance, with darker colors indicating higher sequence counts.

We observed a total of 2,291 *Legionella*-related sequences, which accounted for 0.49% of the total sequences detected in 2019, 0.13% in 2020, and 0.38% in 2022. Most *Legionella*-related sequences were clustered into ASV 9 and ASV 6, which were not closely related to any cultured *Legionella* species (Figure 8), suggesting the presence of potential novel *Legionella* species (Figure 8). Previously, *Legionella* spp. have been detected in aquatic environments, typically in low abundance (Atlas, 1999). They are known to thrive in corroded pipes by forming biofilms (Environmental Protection Agency, 2016) or by scavenging protozoans hosts (Gibbs and Dellinger, 1908). Notably, we did not detect 16S rRNA gene sequences related to *Legionella pneumophila*, which is responsible for over 90% of *Legionella*-related infections (Legionella Control International, 2021). *Legionella pneumophila* can aerosolize and cause Legionnaires’ disease (Fields et al., 2002), which affects approximately 10,000 people annually in the United States with a mortality rate of 10% (Centers for Disease Control and Prevention, 2024). Although *Legionella* sequences were more abundant in 2022 (0.38%) compared to 2020 (0.13%), none were associated with known human pathogens. This outcome was not surprising, as *Legionella* species are known to thrive in the environment using protozoa as hosts.

## 4 Conclusion

The study of Sequiota Spring over three summers provided valuable insights into identifying sources of fecal contamination and the distribution of potential waterborne pathogens in response to repair of leaky old clay sewer pipes. A significant reduction (46 times) in HFIB, particularly *B. dorei*, was observed from 2020 to 2022. The overall 16S rRNA gene abundance of Bacteroidetes-related sequences decreased from 56% of all Bacteroidetes-related sequences retrieved in 2020 to 4% of total Bacteroidetes-related sequences retrieved in in 2022, supporting the decline in human fecal contamination. Likewise, within Enterobacteriaceae-related sequences, *E. kobei* related sequences (a nosocomial pathogen linked to sewage), declined from 13% of total Enterobacteriaceae-related sequences in 2020 to 0.3% of Enterobacteriaceae-related sequences in 2022. Similarly, *Arcobacteraceae* sequences, associated with surface runoff and wastewater contamination, also showed a decrease, particularly *Arcobacter cryaerophilus*, which dropped from 39% of total *Arcobacteraceae* sequences in 2020 to 2.3% in 2022. In contrast, Legionella-related sequences, which thrive in corroded pipes and on protozoan hosts, remained similar throughout the period. We did not detect sequences related to the pathogenic *L. pneumophila*, which is primarily responsible for causing Legionnaires’ disease. Our findings underscore the effectiveness of molecular techniques in identifying problematic infrastructure, mitigating human fecal contamination, and enhancing water quality. Thus, reductions in human fecal contamination should be viewed as closely linked to, but not exclusively caused by, sewer repairs.

## Data Availability

The data presented in this study are publicly available. This data can be found here: https://www.ncbi.nlm.nih.gov/bioproject, accession number PRJNA1338570.

## References

[ref1] Ahmed W. Hughes B. Harwood V. (2016). Current status of marker genes of *Bacteroides* and related taxa for identifying sewage pollution in environmental waters. Water 8:231. doi: 10.3390/w8060231

[ref2] An Y.-J. Breidenbach G. P. (2005). Monitoring *E. coli* and total coliforms in natural spring water as related to recreational mountain areas. Environ. Monit. Assess. 102, 131–137. doi: 10.1007/s10661-005-4691-9 15869182

[ref3] Atlas R. M. (1999). Legionella: from environmental habitats to disease pathology, detection, and control. Environ. Microbiol. 1, 283–293. doi: 10.1046/j.1462-2920.1999.00046.x, PMID: 11207747

[ref4] Bakir M. A. (2006). *Bacteroides dorei* sp. nov., isolated from human faeces. Int. J. Syst. Evol. Microbiol. 56, 1639–1643. doi: 10.1099/ijs.0.64257-0, PMID: 16825642

[ref5] Barka E. A. Vatsa P. Sanchez L. Gaveau-Vaillant N. Jacquard C. Klenk H.-P.. (2015). Taxonomy, physiology, and natural products of actinobacteria. Microbiol. Mol. Biol. Rev. 80, 1–43. doi: 10.1128/mmbr.00019-15 26609051 PMC4711186

[ref6] Baron J. S. Poff N. L. Angermeier P. L. Dahm C. N. Gleick P. H. Hairston N. G.. (2002). Meeting ecological and societal needs for freshwater. Ecol. Appl. 12, 1247–1260. doi: 10.1890/1051-0761(2002)012[1247:MEASNF]2.0.CO;2

[ref7] Berner L. M. (1951). Limnology of the lower Missouri River. Ecology 32, 1–12. doi: 10.2307/1930968

[ref8] Bishop P. K. Misstear B. D. White M. Harding N. J. (1998). Impacts of sewers on groundwater quality. Water Environ. J. 12, 216–223. doi: 10.1111/j.1747-6593.1998.tb00176.x

[ref9] Boyer D. G. Pasquarell G. C. (1999). Agricultural land use impacts on bacterial water quality in a karst groundwater aquifer. J. Am. Water Resour. Assoc. 35, 291–300. doi: 10.1111/j.1752-1688.1999.tb03590.x

[ref10] Buckalew D. W. Hartman L. J. Grimsley G. A. Martin A. E. Register K. M. (2006). A long-term study comparing membrane filtration with Colilert® defined substrates in detecting fecal coliforms and *Escherichia coli* in natural waters. J. Environ. Manag. 80, 191–197. doi: 10.1016/j.jenvman.2005.08.024, PMID: 16338057

[ref11] Buckerfield S. J. Quilliam R. S. Bussiere L. Waldron S. Naylor L. A. Li S.. (2020). Chronic urban hotspots and agricultural drainage drive microbial pollution of karst water resources in rural developing regions. Sci. Total Environ. 744:140898. doi: 10.1016/j.scitotenv.2020.140898, PMID: 32721677

[ref12] Burgmeier N. G. Unger S. D. Meyer J. L. Sutton T. M. Williams R. N. (2011). Health and habitat quality assessment for the eastern hellbender (*Cryptobranchus alleganiensis alleganiensis*) in Indiana, USA. J. Wildl. Dis. 47, 836–848. doi: 10.7589/0090-3558-47.4.836, PMID: 22102654

[ref13] Caporaso J. G. Kuczynski J. Stombaugh J. Bittinger K. Bushman F. D. Costello E. K.. (2010). QIIME allows analysis of high-throughput community sequencing data. Nat. Methods 7, 335–336. doi: 10.1038/nmeth.f.303, PMID: 20383131 PMC3156573

[ref14] Centers for Disease Control and Prevention. (2024). About Legionella. Available online at: https://www.cdc.gov/legionella/about/index.html (Accessed January 1, 2025).

[ref15] Crisler R. M. Hunt M. S. (1952). Recreation regions of Missouri. J. Geogr. 51, 30–39. doi: 10.1080/00221345208982728, PMID: 40989069

[ref16] Diaz P. H. Orsak E. L. Weckerly F. W. Montagne M. A. Alvarez D. A. (2020). Urban stream syndrome and contaminant uptake in salamanders of Central Texas. J. Fish Wildl. Manag. 11, 287–299. doi: 10.3996/032018-JFWM-017

[ref17] Downs G. Upadhyay D. Mandjiny S. Frederick J. Holmes L. (2019). Biological control technology utilizing Heterorhabditis bacteriophora and Steinernema carpocapsae. Int. J. Plant Pathol. 8, 69–76. doi: 10.33687/phytopath.008.02.2890

[ref18] Duchaud E. Rusniok C. Frangeul L. Buchrieser C. Givaudan A. Taourit S.. (2003). The genome sequence of the entomopathogenic bacterium *Photorhabdus luminescens*. Nat. Biotechnol. 21, 1307–1313. doi: 10.1038/nbt886, PMID: 14528314

[ref19] Ehlers R.-U. (2001). Mass production of entomopathogenic nematodes for plant protection. Appl. Microbiol. Biotechnol. 56, 623–633. doi: 10.1007/s002530100711, PMID: 11601608

[ref20] Elmund G. K. Allen M. J. Rice E. W. (1999). Comparison of *Escherichia coli*, total coliform, and fecal coliform populations as indicators of wastewater treatment efficiency. Water Environ. Res. 71, 332–339. doi: 10.2175/106143098x121752

[ref21] Environmental Protection Agency. (2004). Contaminated sediment remediation guidance for hazardous waste sites. Available online at: https://www.epa.gov/sites/default/files/2015-10/documents/csossortc2004_full.pdf (Accessed August 12, 2024).

[ref22] Environmental Protection Agency. (2016). Legionella: drinking water health advisory. Available online at: https://www.epa.gov/sites/default/files/2016-09/documents/legionella_document_master_september_2016_final.pdf (Accessed January 2, 2025).

[ref23] Environmental Protection Agency. (2017). National water quality inventory: report to congress. Available online at: https://www.epa.gov/sites/default/files/2017-12/documents/305brtc_finalowow_08302017.pdf (Accessed August 9, 2024).

[ref24] Environmental Protection Agency. (2024a). Drinking water. Available online at: www.epa.gov/report-environment/drinking-water (Accessed July 8, 2024)

[ref25] Environmental Protection Agency. (2024b). Indicators: cyanobacteria. https://www.epa.gov/national-aquatic-resource-surveys/indicators-cyanobacteria#:~:text=Cyanobacteria%2C%20also%20referred%20to%20as,that%20lead%20to%20cyanobacterial%20blooms (Accessed April 22, 2024).

[ref26] Environmental Protection Agency. (2024c). Effects of nutrient pollution on the economy. https://www.epa.gov/nutrientpollution/effects-economy (Accessed January 9, 2025).

[ref27] Fenner L. Roux V. Mallet M. N. Raoult D. (2005). *Bacteroides massiliensis* sp. nov., isolated from blood culture of a newborn. Int. J. Syst. Evol. Microbiol. 55, 1335–1337. doi: 10.1099/ijs.0.63350-0, PMID: 15879278

[ref28] Fera Figueras M. T. La Camera E. Carbone M. Malara D. Pennisi M. G. (2009). Pet cats as carriers of *Arcobacter* spp. in southern Italy. J. Appl. Microbiol. 106, 1661–1666. doi: 10.1111/j.1365-2672.2008.04133.x 19226387

[ref29] Ferreira S. Queiroz J. A. Oleastro M. Domingues F. C. (2015). Insights in the pathogenesis and resistance of arcobacter: a review. Crit. Rev. Microbiol. 41, 1–20. doi: 10.3109/1040841x.2014.954523 25806423

[ref30] Fields B. S. Benson R. F. Besser R. E. (2002). Legionella and legionnaires’ disease: 25 years of investigation. Clin. Microbiol. Rev. 15, 506–526. doi: 10.1128/cmr.15.3.506-526.2002, PMID: 12097254 PMC118082

[ref31] Figueras M. J. Levican A. Pujol I. Ballester F. Rabada Quilez M. J. Gomez-Bertomeu F. (2014). A severe case of persistent diarrhoea associated with *Arcobacter cryaerophilus* but attributed to Campylobacter sp., and a review of the clinical incidence of Arcobacter spp. New Microbes New Infect. 2, 31–37. doi: 10.1002/2052-2975.35, PMID: 25356338 PMC4184587

[ref32] Fukuyama Y. Inoue M. Omae K. Yoshida T. Sako Y. (2020). Anaerobic and hydrogenogenic carbon monoxide-oxidizing prokaryotes: versatile microbial conversion of a toxic gas into an available energy. Adv. Appl. Microbiol. doi: 10.1016/bs.aambs.2019.12.001, PMID: 32386607

[ref33] García-Aljaro C. Blanch A. R. Campos C. Jofre J. Lucena F. (2018). Pathogens, faecal indicators, and human-specific microbial source-tracking markers in sewage. J. Appl. Microbiol. doi: 10.1111/jam.14112 30244503

[ref34] Gerba C. P. (2015). “Environmentally transmitted pathogens” in Environmental microbiology. eds. Pepper I. L. Gerba C. P. Gentry T. J. (San Diego, CA: Academic Press), 509–550.

[ref35] Giacometti F. Salas-Massó N. Serraino A. Figueras M. J. (2015). Characterization of Arcobacter suis isolated from water buffalo (*Bubalus bubalis*) milk. Food Microbiol. 51, 186–191. doi: 10.1016/j.fm.2015.06.004, PMID: 26187844

[ref36] Gibbs D. Dellinger O. P. (1908). The daily life of *Amoeba proteus*. Am. J. Psychol. 19:232. doi: 10.2307/1412761

[ref37] Green H. C. Dick L. K. Gilpin B. Samadpour M. Field K. G. (2011). Genetic markers for rapid PCR-based identification of gull, Canada goose, duck, and chicken fecal contamination in water. Appl. Environ. Microbiol. 78, 503–510. doi: 10.1128/aem.05734-11 22081573 PMC3255751

[ref38] Green H. C. Haugland R. A. Varma M. Millen H. T. Borchardt M. A. Field K. G.. (2014). Improved HF183 quantitative real-time PCR assay for characterization of human fecal pollution in ambient surface water samples. Appl. Environ. Microbiol. 80, 3086–3094. doi: 10.1128/aem.04137-13, PMID: 24610857 PMC4018914

[ref39] Gücker B. Brauns M. Pusch M. T. (2006). Effects of wastewater treatment plant discharge on ecosystem structure and function of lowland streams. J. N. Am. Benthol. Soc. 25, 313–329. doi: 10.1899/0887-3593(2006)25[313:EOWTPD]2.0.CO;2

[ref40] Harwood V. J. Stoeckel D. M. (2011). “Performance criteria” in Microbial source tracking: Methods, applications, and case studies. eds. Hagedorn C. Blanch A. R. Harwood V. J. (New York, NY: Springer), 7–30.

[ref41] Hatamoto M. Kaneshige M. Nakamura A. Yamaguchi T. (2014). Bacteroides luti sp. nov., an anaerobic, cellulolytic, and xylanolytic bacterium isolated from methanogenic sludge. Int. J. Syst. Evol. Microbiol. 64, 1770–1774. doi: 10.1099/ijs.0.056630-0, PMID: 24556634

[ref42] Hausdorf L. Neumann M. Bergmann I. Sobiella K. Mundt K. Fröhling A.. (2013). Occurrence and genetic diversity of Arcobacter spp. in a spinach-processing plant and evaluation of two Arcobacter-specific quantitative PCR assays. Syst. Appl. Microbiol. 36, 235–243. doi: 10.1016/j.syapm.2013.02.003, PMID: 23561260

[ref43] Hsu T.-T. D. Lee J. (2015). Global distribution and prevalence of *Arcobacter* in food and water. Zoonoses Public Health 62, 579–589. doi: 10.1111/zph.12215, PMID: 26172312

[ref44] Jackson R. B. Carpenter S. R. Dahm C. N. McKnight D. M. Naiman R. J. Postel S. L.. (2001). Water in a changing world. Ecol. Appl. 11, 1027–1045. doi: 10.1890/1051-0761(2001)011[1027:WIACW]2.0.CO;2

[ref45] Janda J. M. Abbott S. L. McIver C. J. (2016). *Plesiomonas shigelloides* revisited. Clin. Microbiol. Rev. 29, 349–374. doi: 10.1128/cmr.00103-15, PMID: 26960939 PMC4786884

[ref46] Kačaroğlu F. (1999). Review of groundwater pollution and protection in karst areas. Water Air Soil Pollut. 113, 337–356. doi: 10.1023/a:1005014532330

[ref47] Katz B. G. Eberts S. M. Kauffman L. J. (2011). Using cl/Br ratios and other indicators to assess potential impacts on groundwater quality from septic systems: a review and examples from principal aquifers in the United States. J. Hydrol. 397, 151–166. doi: 10.1016/j.jhydrol.2010.11.017

[ref48] Kersters K. De Vos P. Gillis M. Swings J. Vandamme P. Stackebrandt E. (2006). “Introduction to the Proteobacteria” in The prokaryotes. eds. Dworkin M. Falkow S. Rosenberg E. Schleifer K. H. Stackebrandt E. (New York, NY: Springer), 3–37.

[ref49] Kincaid J. C. Owen M. R. Pavlowsky R. T. Mirza B. S. (2022). Microbiome of bacterially impaired watersheds: distribution of potential bacterial pathogens. Diversity 14:96. doi: 10.3390/d14020096

[ref50] Kinzelman J. L. Singh A. Ng C. Pond K. R. Bagley R. C. Gradus S.. (2005). Use of IDEXX Colilert-18® and Quanti-tray/2000 as a rapid and simple enumeration method for the implementation of recreational water monitoring and notification programs. Lake Reserv. Manag. 21, 73–77. doi: 10.1080/07438140509354414

[ref51] Koo H. Morrow C. D. (2024). Bacteroidales-specific antimicrobial genes can influence the selection of the dominant fecal strain of Bacteroides vulgatus and *Bacteroides uniformis* from the gastrointestinal tract microbial community. Life 14:555. doi: 10.3390/life14050555, PMID: 38792577 PMC11121782

[ref52] Layton A. Mckay L. Williams D. Garrett V. Gentry R. Sayler G. (2006). Development of *Bacteroides*16S rRNA gene TaqMan-based real-time PCR assays for estimation of total, human, and bovine fecal pollution in water. Appl. Environ. Microbiol. 72, 4214–4224. doi: 10.1128/AEM.01036-05 16751534 PMC1489674

[ref53] Lee C. Kim J. Y. Kim Y. B. Kim J. Ahn S. W. Song H. S.. (2020). A report of 37 unrecorded anaerobic bacterial species isolated from the Geum River in South Korea. J. Species Res. 9, 105–116. doi: 10.12651/JSR.2020.9.2.105

[ref54] Lee S. Suits M. Wituszynski D. Winston R. Martin J. Lee J. (2020). Residential urban stormwater runoff: a comprehensive profile of microbiome and antibiotic resistance. Sci. Total Environ. 723:138033. doi: 10.1016/j.scitotenv.2020.138033, PMID: 32392682

[ref55] Lee L. H. Wu M. Peri A. Chu T.-C. (2014). Method evaluations for *Escherichia coli* and coliforms detection in northern New Jersey water bodies. GSTF J. Biosci. 3:2. doi: 10.7603/s40835-014-0002-y

[ref56] Legionella Control International. (2021). How many Legionella species exist and which ones cause legionnaires’ disease? Available online at: https://legionellacontrol.com/legionella/legionella-species/ (Accessed August 15, 2024).

[ref57] Levican A. Collado L. Figueras M. J. (2013). Arcobacter cloacae sp. nov. and Arcobacter suis sp. nov., two new species isolated from food and sewage. Syst. Appl. Microbiol. 36, 22–27. doi: 10.1016/j.syapm.2012.11.003, PMID: 23265195

[ref58] Levy K. Woster A. P. Goldstein R. S. Carlton E. J. (2016). Untangling the impacts of climate change on waterborne diseases: a systematic review of relationships between diarrheal diseases and temperature, rainfall, flooding, and drought. Environ. Sci. Technol. 50, 4905–4922. doi: 10.1021/acs.est.5b06186, PMID: 27058059 PMC5468171

[ref59] Linton A. H. Hinton M. H. (1988). Enterobacteriaceae associated with animals in health and disease. J. Appl. Bacteriol. 65, 71S–85S. doi: 10.1111/j.1365-2672.1988.tb04646.x, PMID: 3142053

[ref60] Lu J. Santo Domingo J. W. Hill S. Edge T. A. (2009). Microbial diversity and host-specific sequences of Canada goose feces. Appl. Environ. Microbiol. 75, 5919–5926. 19633110 10.1128/AEM.00462-09 PMC2747875

[ref61] Ludden C. Reuter S. Judge K. Gouliouris T. Blane B. Coll F.. (2017). Sharing of carbapenemase-encoding plasmids between Enterobacteriaceae in UK sewage uncovered by MinION sequencing. Microb. genomics 3:e000114. doi: 10.1099/mgen.0.000114 PMC5605956 29026655

[ref62] Mayhood P. Mirza B. S. (2021). Soybean root nodule and rhizosphere microbiome: distribution of rhizobial and nonrhizobial endophytes. Appl. Environ. Microbiol. 87:e02884, –20. doi: 10.1128/AEM.02884-20, PMID: 33674438 PMC8117765

[ref63] McCance W. Jones O. A. H. Cendón D. I. Edwards M. Surapaneni A. Chadalavada S.. (2020). Combining environmental isotopes with contaminants of emerging concern (CECs) to characterise wastewater-derived impacts on groundwater quality. Water Res. 182:116036. doi: 10.1016/j.watres.2020.116036, PMID: 32645458

[ref64] Miller M. L. Koburger J. A. (1985). *Plesiomonas shigelloides*: An opportunistic food and waterborne pathogen. J. Food Prot. 48, 449–457. doi: 10.4315/0362-028x-48.5.449, PMID: 30943637

[ref65] Mirza B. S. Sorensen D. L. McGlinn D. J. Dupont R. R. McLean J. E. (2017). *Dehalococcoides* and general bacterial ecology of differentially trichloroethene dechlorinating flow-through columns. Appl. Microbiol. Biotechnol. 101, 4799–4813. doi: 10.1007/s00253-017-8180-1, PMID: 28213734

[ref66] Müller E. Hotzel H. Ahlers C. Hänel I. Tomaso H. Abdel-Glil M. Y. (2020). Genomic analysis and antimicrobial resistance of Aliarcobacter cryaerophilus strains from German water poultry. Front. Microbiol. 11:1549. doi: 10.3389/fmicb.2020.01549, PMID: 32754133 PMC7365950

[ref67] Newman L. Duffus A. L. J. Lee C. (2016). Using the free program MEGA to build phylogenetic trees from molecular data. Am. Biol. Teach. 78, 608–612. doi: 10.1525/abt.2016.78.7.608

[ref68] Nieva-Echevarria B. Martinez-Malaxetxebarria I. Girbau C. Alonso R. Fernández-Astorga A. (2013). Prevalence and genetic diversity of Arcobacter in food products in the north of Spain. J. Food Prot. 76, 1447–1450. doi: 10.4315/0362-028x.jfp-13-014, PMID: 23905804

[ref69] Nishiyama T. Ueki A. Kaku N. Watanabe K. Ueki K. (2009). *Bacteroides graminisolvens* sp. nov., a xylanolytic anaerobe isolated from a methanogenic reactor treating cattle waste. Int. J. Syst. Evol. Microbiol. 59, 1901–1907. doi: 10.1099/ijs.0.008268-0, PMID: 19567576

[ref70] Nshimyimana J. P. Ekklesia E. Shanahan P. Chua L. H. C. Thompson J. R. (2014). Distribution and abundance of human-specific Bacteroides and relation to traditional indicators in an urban tropical catchment. J. Appl. Microbiol. 116, 1369–1383. doi: 10.1111/jam.12455, PMID: 24460587 PMC4271309

[ref71] Osunmakinde C. O. Selvarajan R. Mamba B. B. Msagati T. A. M. (2019). Profiling bacterial diversity and potential pathogens in wastewater treatment plants using high-throughput sequencing analysis. Microorganisms 7:506. doi: 10.3390/microorganisms7110506, PMID: 31671809 PMC6921039

[ref72] Owen M. R. Mirza B. S. Pursley T. J. Pavlowsky R. T. (2021). Bacteria source tracking assessment of Sequiota spring. Springfield, MO, USA: Missouri State University.

[ref73] Owen M. R. Mirza B. S. Roman G. F. Kincaid J. C. Pavlowsky R. T. (2019). Bacteria source tracking to support watershed planning, Pearson Creek, Greene County, Missouri. OEWRI technical reports, 25.

[ref74] Pronk M. Goldscheider N. Zopfi J. (2007). Particle-size distribution as indicator for fecal bacteria contamination of drinking water from karst springs. Environ. Sci. Technol. 41, 8400–8405. doi: 10.1021/es071976f, PMID: 18200870

[ref75] R Development Core Team (2024). R: A language and environment for statistical computing. Vienna, Austria: R Foundation for Statistical Computing.

[ref76] Ranjani A. Dhanasekaran D. Gopinath P. M. (2016). “An introduction to Actinobacteria” in Actinobacteria - basics and biotechnological applications. eds. Dhanasekaran D. Jiang Y. (Rijeka: InTechOpen), 3–37.

[ref77] Reynolds J. H. Barrett M. H. (2003). A review of the effects of sewer leakage on groundwater quality. Water Environ. J. 17, 34–39. doi: 10.1111/j.1747-6593.2003.tb00428.x

[ref78] Rice L. B. (2008). Federal funding for the study of antimicrobial resistance in nosocomial pathogens: no ESKAPE. J. Infect. Dis. 197, 1079–1081. doi: 10.1086/533452, PMID: 18419525

[ref79] Romaní-Pérez M. López-Almela I. Bullich-Vilarrubias C. Evtoskí Z. Benitez-Páez A. Sanz Y. (2024). *Bacteroides uniformis* CECT 7771 requires adaptive immunity to improve glucose tolerance but not to prevent body weight gain in diet-induced obese mice. Microbiome 12:103. doi: 10.1186/s40168-024-01810-3, PMID: 38845049 PMC11155119

[ref80] Rstudio Team. (2024). RStudio: Integrated development for R (version 4.4.1) [software]. RStudio, PBC, Boston, MA, USA. Available online at: http://www.rstudio.com/ (Accessed August 2, 2024).

[ref81] Seurinck S. Defoirdt T. Verstraete W. Siciliano S. D. (2005). Detection and quantification of the human-specific HF183 Bacteroides 16S rRNA genetic marker with real-time PCR for assessment of human faecal pollution in freshwater. Environ. Microbiol. 7, 249–259. doi: 10.1111/j.1462-2920.2004.00702.x, PMID: 15658992

[ref82] Spain A. M. Krumholz L. R. Elshahed M. S. (2009). Abundance, composition, diversity, and novelty of soil Proteobacteria. ISME J. 3, 992–1000. doi: 10.1038/ismej.2009.43, PMID: 19404326

[ref83] Sterk A. Schijven J. de Nijs T. de Roda Husman A. M. (2013). Direct and indirect effects of climate change on the risk of infection by water-transmitted pathogens. Environ. Sci. Technol. 47, 12648–12660. doi: 10.1021/es403549s, PMID: 24125400

[ref84] Teixeira P. Dias D. Costa S. Brown B. Silva S. Valério E. (2020). *Bacteroides* spp. and traditional fecal indicator bacteria in water quality assessment – an integrated approach for hydric resources management in urban centers. J. Environ. Manag. 271:110989. doi: 10.1016/j.jenvman.2020.110989, PMID: 32579514

[ref85] Thomas F. Hehemann J.-H. Rebuffet E. Czjzek M. Michel G. (2011). Environmental and gut Bacteroidetes: the food connection. Front. Microbiol. 2:93. doi: 10.3389/fmicb.2011.00093, PMID: 21747801 PMC3129010

[ref86] Tseng H. K. Liu C. P. Li W. C. Su S. C. Lee C. M. (2002). Characteristics of *Plesiomonas shigelloides* infection in Taiwan. J. Microbiol. Immunol. Infect. 35, 47–52. 11950120

[ref87] Vineyard J.D. Feder G.L. (1982). Springs of Missouri. Missouri department of natural resources, division of geology and land survey. Water resources report #29. U.S. Geological Survey. Available online at: https://share.mo.gov/nr/mgs/MGSData/Books/Water%20Resources/Springs%20of%20Missouri/WR29revised.pdf (Accessed March 15, 2024).

[ref88] Waksman S. A. (1931). Decomposition of the various chemical constituents of complex plant materials by pure cultures of fungi and bacteria. Arch. Microbiol. 2, 136–154. doi: 10.1007/bf00446500

[ref89] Whitton B. A. Potts M. (2002). The ecology of Cyanobacteria: Their diversity in time and space. Dordrecht: Springer.

[ref90] Withers P. J. Jordan P. May L. Jarvie H. P. Deal N. E. (2014). Do septic tank systems pose a hidden threat to water quality? Front. Ecol. Environ. 12, 123–130. doi: 10.1890/130131

[ref91] Wright Water Engineers. (2001). Southern Hills lakes preliminary evaluation and management plan: summary report prepared for the City of Springfield. Available online at: https://www.springfieldmo.gov/DocumentCenter/View/3235/Southern-Hills-Report-PDF?bidId= (Accessed March 15, 2024).

[ref92] Yu S. Y. Kim J.-S. Oh B. S. Park S.-H. Kang S. W. Park J.-E.. (2019). Bacteroides faecalis sp. nov., isolated from human faeces. Int. J. Syst. Evol. Microbiol. 69, 3824–3829. doi: 10.1099/ijsem.0.003690, PMID: 31511127

[ref93] Zhang Y. Kelly W. R. Panno S. V. Liu W.-T. (2014). Tracing fecal pollution sources in karst groundwater by Bacteroidales genetic biomarkers, bacterial indicators, and environmental variables. Sci. Total Environ. 490, 1082–1090. doi: 10.1016/j.scitotenv.2014.05.086, PMID: 24922611

[ref94] Zhou K. Yu W. Cao X. Shen P. Lu H. Luo Q.. (2017). Characterization of the population structure, drug resistance mechanisms, and plasmids of the community-associated *Enterobacter cloacae* complex in China. J. Antimicrob. Chemother. 73, 66–76. doi: 10.1093/jac/dkx361 29088362

[ref95] Zwart G. Crump B. C. Kamst-van A. M. P. Hagen F. Han S. (2002). Typical freshwater bacteria: an analysis of available 16S rRNA gene sequences from plankton of lakes and rivers. Aquat. Microb. Ecol. 28, 141–155. doi: 10.3354/ame028141

